# Improvement of the in vitro fertilization and embryo development using frozen–thawed spermatozoa of microminipigs

**DOI:** 10.5194/aab-64-265-2021

**Published:** 2021-06-16

**Authors:** Zhao Namula, Yasuhiro Isumi, Yoko Sato, Quynh Anh Le, Qingyi Lin, Koki Takebayashi, Maki Hirata, Fuminori Tanihara, Chommanart Thongkittidilok, Takeshige Otoi

**Affiliations:** 1 Faculty of Veterinary Science, Guangdong Ocean University, Zhanjiang, China; 2 Faculty of Bioscience and Bioindustry, Tokushima University, Tokushima, Japan; 3 School of Biological Science, Tokai University, Sapporo, Japan; 4 Akkhraratchakumari Veterinary College, Walailak University, Nakorn Sri Thammarat, Thailand

## Abstract

This study aimed to compare the quality and the penetration ability of frozen–thawed spermatozoa from three microminipigs and Large White boars and to evaluate the effects of caffeine and heparin as well as the sperm–oocyte co-incubation length on the fertilization and embryonic development in vitro. Results showed that the fertilization rates of spermatozoa from three microminipig boars were significantly lower than those of a Large White boar. In the post-thaw spermatozoa from one of three microminipig boars, the sperm quality, penetration ability, and the oocyte development after in vitro fertilization were significantly lower than those of the spermatozoa from other boars. The caffeine supplementation in the fertilization media increased the rates of fertilization and blastocyst formation for the microminipig spermatozoa with low sperm quality. In addition to caffeine supplementation, the rates of fertilization and blastocyst formation after using microminipig spermatozoa were significantly higher with a 10 h sperm–oocyte co-incubation than with 3 h of co-incubation length. Our results indicate that the differences between the males and the breed influence the quality and fertility of frozen–thawed boar spermatozoa. In conclusion, the presence of caffeine in the in vitro fertilization (IVF) medium and adequate length of sperm–oocyte co-incubation may have beneficial effects for improving IVF results when using microminipig spermatozoa with low quality.

## 1 Introduction

Microminipigs have a tiny body size (7 to 8 kg for a 6-month­old mature pig), which provides many advantages for laboratory use, including ease of handling, small rearing spaces, and low doses of test substances (Kaneko et al., 2011). A cross between microminipigs and the large western pig is expected to result in a smaller pig than the western pig. This method is useful, for example, when miniaturizing genetically modified animals produced from large western pigs. However, the natural mating of microminipigs with the large western pig is difficult due to differences in size. Therefore, the use of in vitro fertilization (IVF) is one of the useful tools for the production of a crossbreed using frozen–thawed spermatozoa of microminipigs as an alternative to natural mating.

Cryopreserved semen allows the use of single ejaculates for the repeated production of the crossbreed, potentially improving IVF consistency by eliminating inter-ejaculate variability observed with fresh semen. However, the freezing and thawing processes result in compromised sperm function and IVF success. It has been suggested that various circumstances are associated with the low fertility of frozen–thawed boar spermatozoa, which include damage to the spermatozoa during the freeze–thaw process (Yeste, 2016). It is well known that individual differences in the susceptibility of sperm cells to low temperatures between boars exist (Waterhouse et al., 2006). Moreover, the response of sperm to cryopreservation and the motility of frozen–thawed spermatozoa may vary between different breeds. Therefore, it is crucial to identify the differences between the males and different breeds in the fertility of frozen–thawed boar spermatozoa for the improvement of IVF results.

Mammalian spermatozoa are not immediately capable of fertilizing an egg until they undergo capacitation, either within the female reproductive tract or in a suitable medium in vitro. Caffeine is a well-known non-specific inhibitor of phosphodiesterase and a potent capacitation inducer of porcine spermatozoa (Funahashi et al., 2000). Induction of capacitation and the acrosome reaction by caffeine results in a high sperm penetration rate, indicating that several immotile spermatozoa have the potential to become motile as a result of caffeine treatment (Corcuera et al., 2007; Funahashi and Nagai, 2001). By contrast, heparin is the most potent inducer of bovine sperm capacitation and the acrosome reaction (Parrish, 2014). In pigs, heparin has been reported to enhance in vitro capacitation of porcine sperm as assessed using chlortetracycline staining (Dapino et al., 2006). Therefore, there is a possibility that capacitating agents, such as caffeine and heparin, may improve the fertility of frozen–thawed boar spermatozoa with low quality.

This study was designed to evaluate the quality, penetration ability, and oocyte development of frozen–thawed spermatozoa from microminipigs. To improve the poor penetration ability of microminipig spermatozoa, we evaluated the effects of caffeine and heparin supplementation during IVF and sperm–oocyte co-incubation length on the fertilization and development of porcine oocytes.

## 2 Materials and methods

### 2.1 Animal care

All animal care and the experiments were performed following the Guide for the Care and Use of Laboratory Animals prepared by the Institutional Animal Care and Use Committee of Tokushima University.

### 2.2 Semen collection and cryopreservation

Semen collection and cryopreservation were performed as previously described (Namula et al., 2018). Briefly, semen was collected from three microminipig boars (1.5–2 years old) with unknown results of sperm parameter, fertilization, and artificial insemination (AI) and from a Large White boar (3 years old) with known in vitro fertilization results but unknown sperm parameter and AI results, using the “gloved-hand” technique. Semen samples were diluted with the first extender, which consisted of 0.4 mg/mL D-fructose (Sigma-Aldrich, St. Louis, MO, USA), 2.9 mg/mL Tris (hydroxymethyl) aminomethane (Tris; Sigma-Aldrich), 1.59 mg/mL citric acid monohydrate (Wako Pure Chemical Industries, Osaka, Japan), 0.2 mg/mL amikacin sulfate (Meiji, Tokyo, Japan), and 20 % (v/v) egg yolk in distilled water. The semen samples were cooled to 5 ${}^{\circ }$C from room temperature and then diluted to a final concentration of 2 × 10${}^{8}$ cells/mL with the second extender (the first extender supplemented with 3 % (v/v) glycerol (Wako Pure Chemical Industries) and 0.74 % (v/v) EQUEX STM paste (Miyazaki Kagaku, Tokyo, Japan)). The spermatozoa were loaded into the 0.25 mL French straws and frozen in liquid nitrogen. On the day of the examination, the straw was immediately submerged into a 38 ${}^{\circ }$C water bath for 10 s for thawing.

### 2.3 Assessment of motility and quality of sperm

The motility and quality of frozen–thawed spermatozoa from three microminipig boars and a Large White boar were examined. Briefly, motility analyses of frozen–thawed spermatozoa were performed using the computer-assisted sperm analysis system (sperm class analyser: SCA v.4.2; MICROPTIC, Barcelona, Spain). The motility analysis was based on the examination of 25 consecutive digitized images obtained from three to five fields using a × 10 phase contrast objective, and at least 500 spermatozoa per sample were analysed using an image capture speed of 40 ms. Viability, plasma membrane integrity, and acrosome integrity analyses were conducted using a live–dead stain combination (SYBR-14/propidium iodide (PI), LIVE/DEAD Sperm Viability Kit; Molecular Probes, Inc., Eugene, OR, USA), the hypo-osmotic swelling test (Ahmad et al., 2003), and fluorescein isothiocyanate-labelled peanut agglutinin (FITC-PNA; Vector Laboratories, Inc., Burlingame, CA, USA), respectively, according to the methods described by Wittayarat et al. (2012).

### 2.4 Assessment of penetration ability of sperm and oocyte development

Oocyte collection, in vitro maturation, fertilization, and embryo culture were carried out as described previously (Nishio et al., 2018). Briefly, we obtained pig ovaries from prepubertal crossbred gilts (Landrace × Large White × Duroc breeds) at a local slaughterhouse. Cumulus–oocyte complexes (COCs) with a uniform ooplasm and compact cumulus cell mass were collected by ovary slicing and then cultured in a maturation medium at 39 ${}^{\circ }$C in a humidified incubator containing 5 % CO${}_{2}$. The maturation medium consisted of tissue culture medium 199 with Earle's salts (TCM 199; Invitrogen Co., Carlsbad, CA, USA), supplemented with 10 % (v/v) porcine follicular fluid, 0.6 mM cysteine (Sigma-Aldrich, St. Louis, MO, USA), 50 µM sodium pyruvate (Sigma-Aldrich), 2 mg/mL D-sorbitol (Wako Pure Chemical Industries Ltd., Osaka, Japan), 50 µM β-mercaptoethanol (Wako Pure Chemical Industries Ltd.), 10 IU/mL equine chorionic gonadotropin (Kyoritu Seiyaku, Tokyo, Japan), 10 IU/mL human chorionic gonadotropin (Kyoritu Seiyaku), and 50 µg/mL gentamicin (Sigma-Aldrich). After maturation for 20–22 h, the COCs were cultured for an additional 24 h in the maturation medium without hormones under the same conditions.

For IVF, frozen–thawed spermatozoa from same batch of each boar were transferred into 6 mL of porcine fertilization medium (PFM; Research Institute for Functional Peptides Co., Yamagata, Japan) and washed by centrifuging at 500 × g for 5 min. The pelleted spermatozoa were resuspended in PFM, and then the oocytes cultured in the maturation medium (around 45 oocytes per repeat in each boar group) were transferred to the sperm-containing PFM and co-incubated for 5 h in a humidified incubator containing 5 % CO${}_{2}$, 5 % O${}_{2}$, and 90 % N${}_{2}$ at 39 ${}^{\circ }$C.

Some zygotes were mounted on glass slides 10 h after insemination and fixed with acetic acid : ethanol (1:3, v/v) for 48–72 h to assess oocyte fertilization. The fixed zygotes were stained with acetic orcein (1 % orcein in 45 % acetic acid) and examined using a phase contrast microscope. Oocytes containing both female and male pronuclei were considered as fertilized and were categorized as normal or polyspermic based on the number of swollen sperm heads and/or pronuclei in the cytoplasm (Do et al., 2015).

After IVF, the zygotes were washed with pig zygote medium (PZM-5; Research Institute for Functional Peptides Co.) and cultured continuously at 39 ${}^{\circ }$C in a humidified incubator containing 5 % CO${}_{2}$, 5 % O${}_{2}$, and 90 % N${}_{2}$. Embryos cultured for 3 d were subsequently incubated in porcine blastocyst medium (PBM; Research Institute for Functional Peptides Co.) for 4 d. The cleavage and blastocyst formation were evaluated 3 and 7 d after IVF, respectively.

### 2.5 Evaluation of sperm concentration

COCs that were matured in vitro for 44 h were co-incubated for 5 h with two different concentrations (1.0 × 10${}^{6}$ and 2.0 × 10${}^{6}$ cells/mL) of frozen–thawed spermatozoa from microminipig and Large White boars to evaluate the effects of sperm concentration on the penetration ability of the sperm and oocyte development after IVF.

### 2.6 Evaluation of caffeine and heparin supplementation and sperm–oocyte co-incubation length

The assessment of quality and penetration ability of frozen–thawed spermatozoa revealed that one of the three microminipig spermatozoa had lower motility, quality, penetration ability, and embryo development after IVF. Moreover, the sperm concentration did not affect the fertilization and embryo development in any of the males. Therefore, to improve the reduced fertility of the microminipig spermatozoa, we evaluated the effects of caffeine (3 mM) and heparin (10 IU/mL) supplementation, either alone or in combination, in the fertilization medium on the fertilization and development of porcine oocytes after IVF using 1.0 × 10${}^{6}$ cells/mL of spermatozoa. Moreover, we examined the effects of sperm–oocyte co-incubation length (3–20 h) on the fertilization and development of porcine oocytes fertilized with frozen–thawed spermatozoa, while supplementing them with caffeine concentration that we found to be suitable for the microminipig spermatozoa with low quality.

### 2.7 Statistical analysis

Each experiment was repeated four to six times. Data on sperm quality and embryonic development were evaluated using analysis of variance (ANOVA) using the general linear model (GLM) procedure of the SAS software (SAS for Windows, version 9.1, SAS Institute Japan, Tokyo, Japan). Normality was evaluated with a Kolmogorov–Smirnov test with a 5 % significance level. Arcsine square root transformation was carried out when distribution was not normal. The statistical model included the type of sperm concentration, the boar, and the two-way interactions. Non-significant interactions were excluded from the model. Differences with a probability value (P) of 0.05 or less were regarded as significant.

## 3 Results

### 3.1 Evaluation of quality and penetration ability of frozen–thawed spermatozoa from microminipig and Large White boars

**Figure 1:**
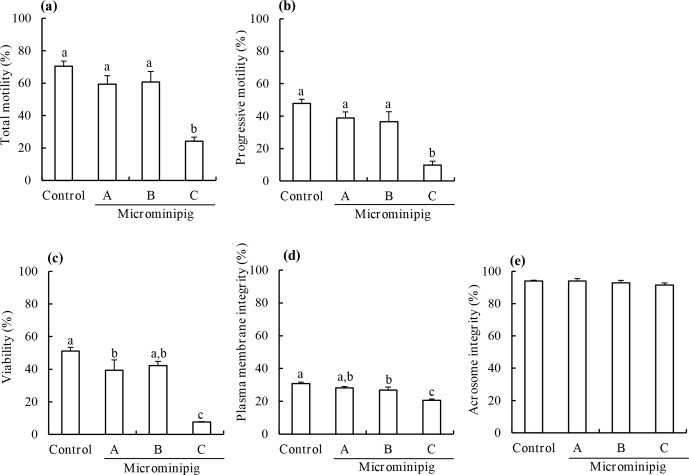
The total motility **(a)**, progressive motility **(b)**, viability **(c)**, plasma membrane integrity **(d)**, and acrosomal integrity **(e)** of frozen–thawed spermatozoa collected from three microminipig boars. As a control, spermatozoa were collected from a Large White boar. Data are presented as mean ± standard error of mean in four replicate experiments, using the same batch of each boar. **(a–c)** Bars with different letters differ significantly (P<0.05).

The motility, viability, plasma membrane integrity, and acrosomal integrity of frozen–thawed spermatozoa from three microminipig boars and a Large White boar are shown in Fig. 1. The total and progressive motility, viability, and plasma membrane integrity of post-thaw spermatozoa from the microminipig boar C significantly decreased compared with those of other boars (P<0.05). There was no significant difference in the acrosome integrity of post-thaw spermatozoa, irrespective of the boar (P>0.05).

The effects of sperm concentration and different boars on the fertilization and development of porcine oocytes fertilized with frozen–thawed spermatozoa are shown in Table 1. There were no differences in the rates of total and monospermic fertilization and blastocyst formation between two sperm concentrations, irrespective of the boar. However, the rates of total fertilization and blastocyst formation were significantly lower (P<0.05) in spermatozoa from microminipig boar C than in spermatozoa from other boars. Moreover, the fertilization rate of spermatozoa from microminipig boars were significantly lower (P<0.05) than that of spermatozoa from the Large White boar, irrespective of sperm concentration.

**Table 1:** Effects of sperm concentration and different boars on the fertilization and development of porcine oocytes fertilized with frozen–thawed spermatozoa from microminipigs.*

Boar**	Sperm	No.	No. (%) of oocytes***		No. of	No. (%) of embryos****	
	concentration	of examined	Fertilized	Monospermy	examined	Cleaved	Developed to
	($\times 10^{6}$ cells/mL)	oocytes			embryos		blastocysts
Control	1.0	110	76.1 ± 5.4${}^{\text{a}}$	55.8 ± 6.0${}^{\text{b}}$	240	84.4 ± 1.1${}^{\text{a, c}}$	20.8 ± 2.1${}^{\text{a}}$
	2.0	118	76.0 ± 4.2${}^{\text{a}}$	48.9 ± 4.9${}^{\text{b}}$	222	88.3 ± 1.3${}^{\text{a, c}}$	22.9 ± 1.2${}^{\text{a}}$
Microminipig	1.0	127	58.8 ± 6.3${}^{\text{b}}$	61.8 ± 3.4${}^{\text{b}}$	249	82.8 ± 2.2${}^{\text{c. d}}$	19.1 ± 4.8${}^{\text{a}}$
A	2.0	115	61.5 ± 4.9${}^{\text{b}}$	60.0 ± 5.0${}^{\text{b}}$	243	88.7 ± 2.9${}^{\text{a}}$	18.9 ± 4.1${}^{\text{a}}$
Microminipig	1.0	114	56.9 ± 5.3${}^{\text{b}}$	67.2 ± 4.8${}^{\text{b}}$	263	82.1 ± 3.5${}^{\text{c, e}}$	18.1 ± 3.1${}^{\text{a}}$
B	2.0	122	55.8 ± 6.6${}^{\text{b}}$	64.1 ± 4.2${}^{\text{b}}$	230	83.3 ± 2.6${}^{\text{a, c}}$	16.3 ± 4.8${}^{\text{a}}$
Microminipig	1.0	125	12.3 ± 3.4${}^{\text{c}}$	97.1 ± 2.9${}^{\text{a}}$	225	74.8 ± 3.5${}^{\text{b, d, e}}$	2.2 ± 1.7${}^{\text{b}}$
C	2.0	118	16.6 ± 3.2${}^{\text{c}}$	90.0 ± 10.0${}^{\text{a}}$	245	74.2 ± 3.9${}^{\text{b, e}}$	0.8 ± 0.5${}^{\text{b}}$

* Five replicated trials were carried out. Percentages are expressed as mean ± standard error of mean.** As control, spermatozoa were collected from a Large White boar. *** The monospermic fertilization rate was defined as a ratio of the number of monospermic oocytes to the total number of fertilized oocytes.**** The blastocyst formation rate was defined as a ratio of the number of blastocysts to the total number of inseminated oocytes. ${}^{\text{a–e}}$ Values with different superscripts in the same column differ significantly (P<0.05).

### 3.2 Effects of caffeine and heparin supplementation and sperm–oocyte co-incubation length

The effect of caffeine and heparin supplementation on the fertilization and oocyte development of the spermatozoa from microminipig C are shown in Table 2. Supplementation of caffeine alone gave significantly higher rates of total fertilization and blastocyst formation but not of cleavage rates compared with others (P<0.05). In contrast, the supplementation of heparin alone or in combination with caffeine had no effects on the fertilization and oocyte development.

**Table 2:** Effects of caffeine and heparin supplementation, either alone or in combination, on the fertilization and development of porcine oocytes fertilized with frozen–thawed spermatozoa from microminipig C*.

Group**	No. of	No. (%) of oocytes***		No. of	No. (%) of embryos****	
	examined	Fertilized	Monospermy	examined	Cleaved	Developed to
	oocytes			embryos		blastocysts
Control	124	8.9 ± 1.3${}^{\text{b}}$	100${}^{\text{a}}$	246	80.8 ± 1.5	1.9 ± 1.0${}^{\text{b}}$
Caffeine	116	25.8 ± 4.1${}^{\text{a}}$	83.0 ± 5.8${}^{\text{b}}$	260	85.4 ± 2.8	5.5 ± 1.7${}^{\text{a}}$
Heparin	127	7.7 ± 1.2${}^{\text{b}}$	100${}^{\text{a}}$	251	84.4 ± 1.6	1.8 ± 0.9${}^{\text{b}}$
Combination	117	11.2 ± 1.8${}^{\text{b}}$	100${}^{\text{a}}$	267	83.6 ± 1.3	1.9 ± 0.8${}^{\text{b}}$

* Six replicated trials were carried out. Percentages are expressed as mean ± standard error of mean. The concentration of spermatozoa used for in vitro fertilization (IVF) was 1 × 10${}^{6}$ sperm/mL.** As control, the cumulus–oocyte complexes were co-incubated with spermatozoa without caffeine (3 mM) and heparin (10 IU/mL). *** The monospermic fertilization rate was defined as a ratio of the number of monospermic oocytes and the total number of fertilized oocytes.**** The blastocyst formation rate was defined as a ratio of the number of blastocysts to the total number of inseminated oocytes. ${}^{\text{a,b}}$ Values with different superscripts in the same column differ significantly (P<0.05).

The effects of sperm–oocyte co-incubation length on the fertilization and oocyte development using spermatozoa from microminipig C are shown in Table 3. The rates of total fertilization, cleavage, and blastocyst formation were significantly higher (P<0.05) after a 10 h co-incubation than a 3 h co-incubation. However, an increase in co-incubation length to 20 h did not enhance the sperm penetration ability and oocyte development. The rate of monospermic fertilization decreased when co-incubation lasted for more than 5 h.

**Table 3:** Effects of sperm–oocyte co-incubation length on the fertilization and development of porcine oocytes fertilized with frozen–thawed spermatozoa from microminipig C*.

IVF length (h)	No. of	No. (%) of oocytes***		No. of	No. (%) of embryos****	
	examined	Fertilized	Monospermy	examined	Cleaved	Developed to
	oocytes			embryos		blastocysts
3	113	19.5 ± 3.6${}^{\text{b}}$	93.9 ± 3.9${}^{\text{a}}$	276	78.2 ± 2.0${}^{\text{b}}$	3.6 ± 0.9${}^{\text{b}}$
5	112	28.2 ± 3.5${}^{\text{a, b}}$	64.0 ± 5.7${}^{\text{b}}$	289	85.5 ± 2.0${}^{\text{a,}}$	6.9 ± 1.0${}^{\text{a, b}}$
10	121	35.7 ± 3.1${}^{\text{a}}$	63.8 ± 3.2${}^{\text{b}}$	275	85.4 ± 1.8${}^{\text{a}}$	10.2 ± 1.9${}^{\text{a}}$
20	112	38.0 ± 5.8${}^{\text{a}}$	58.7 ± 4.7${}^{\text{b}}$	276	81.5 ± 1.5${}^{\text{a, b}}$	6.7 ± 1.3${}^{\text{a, b}}$

* Six replicated trials were carried out. Percentages are expressed as mean ± standard error of mean. The concentrations of spermatozoa and caffeine used for in vitro fertilization (IVF) were 1 × 10${}^{6}$ sperm/mL and 3 mM, respectively.** The monospermic fertilization rate was defined as a ratio of the number of monospermic oocytes and the total number of fertilized oocytes.*** The blastocyst formation rate was defined as a ratio of the number of blastocysts to the total number of inseminated oocytes. ${}^{\text{a,b}}$ Values with different superscripts in the same column differ significantly (P<0.05).

## 4 Discussion

Cryopreservation causes damage to spermatozoa, leading to changes in membrane lipid composition, acrosome status, sperm motility, and viability (Gangwar et al., 2018). The most obvious sign of cold shock is the loss of motility which is not regained when the semen is thawed (White, 1993). In the present study, a Large White boar with known in vitro fertilization results was used as a control; it has been bred on the same farm as three microminipig boars. We observed that in the post-thaw spermatozoa from microminipig boar C, there was a significant decrease not only in the motility but also in the other quality parameters (viability and plasma membrane integrity), the penetration ability, and the oocyte development after IVF. Although the total and progressive motility of other microminipig boars (A and B) were similar to those of the Large White boar (control), the total fertilization rate of spermatozoa from the microminipig boars were lower. It is known that there are variations in sperm quality among boars and ejaculates of the same boar (Barbas and Mascarenhas, 2009). Especially, the different males but not the breed has been suggested to be the main factor influencing the variability in sperm cryosurvival (Fraser et al., 2014). However, we found that there were differences in the fertility of frozen–thawed boar spermatozoa in both different males and the breed. It has been suggested that there are differences in the composition of fatty acids in the sperm cell membrane among breeds, influencing capacitation and capability for cryopreservation (Waterhouse et al., 2004, 2006). Therefore, the breed-specific difference observed in the present study may result in part from the different composition of fatty acids in the sperm cell membrane.

The most accurate indication of boar semen quality is viable pregnancies and offspring following AI, but this is not feasible as a routine method of sperm fertility assay (Larsson and Rodriguez-Martinez, 2000). Therefore, IVF seems to be a helpful method for assessing the penetration ability of sperm (Xu et al., 1998). In the present study, we investigated whether the concentration of frozen–thawed spermatozoa from each boar, the caffeine and heparin supplementation, and the sperm–oocyte co-incubation length during IVF have any effects on the fertilization and oocyte development. Moreover, when the concentration of frozen–thawed spermatozoa from each boar was evaluated, doubling the sperm concentration did not affect the fertilization and oocyte development, irrespective of different males and breed. However, supplementation of caffeine at least had a beneficial effect on the penetration ability and blastocyst formation of the microminipig spermatozoa with low sperm quality. Caffeine has been used to induce sperm capacitation and spontaneous acrosome reaction in porcine IVF (Funahashi and Nagai, 2001; Nagai et al., 2006). The induction of capacitation and/or acrosome reaction by caffeine results in an increased rate of sperm penetration into porcine oocytes in vitro (Funahashi and Nagai, 2001). However, a high incidence of polyspermic penetration has been observed in almost all porcine IVF trials with fresh or frozen–thawed spermatozoa that contain caffeine in the fertilization medium (Funahashi and Day, 1997; Nagai and Moor, 1990). Our results similarly showed that caffeine supplementation decreased the rate of monospermic fertilization; however, the supplementation increased the rate of total fertilization and blastocyst formation, although there were no effects on the cleavage rates. In contrast, the supplementation of heparin was not effective for the improvement of the penetration ability of low-quality frozen–thawed spermatozoa. Moreover, the combination of heparin and caffeine resulted in lower efficiency than caffeine alone for the penetration ability and blastocyst formation. The reason for non-effective supplementation of heparin on the fertilization remains unclear. However, it has been suggested that DNA breaks and overoxidation of thiol groups in protamines can play an important role in chromatin destabilization of frozen–thawed boar spermatozoa (Cordova-Izquierdo et al., 2006)) and the less stable chromatin after heparin treatment is related to more damaged DNA (Corcuera et al., 2007). Therefore, our results indicate that the supplementation of heparin during porcine IVF may not contribute to the improvement of the penetration ability of spermatozoa with poor quality.

The length of sperm–oocyte co-incubation during insemination is one of the factors that influence IVF results (Coy et al., 1993; Gil et al., 2007). In the present study, we found that the penetration and blastocyst formation were improved by exposing the oocytes to the spermatozoa for 10 h in the IVF medium containing caffeine. Our results are in agreement with the experiment of Nguyen et al. (2018), who reported that the combination of an elevated caffeine concentration in an IVF medium and an extended interval of IVF with an optimized concentration is a potent way to improve the fertilization results for porcine spermatozoa with low fertility. However, the increase in co-incubation length to more than 5 h decreased the rate of monospermic fertilization. A longer co-incubation of IVF was effective for zygote production, which increased the penetration rate, resulting in high zygote production rate, although the monospermy rate decreased. It has been suggested that a transient co-incubation of porcine oocytes with spermatozoa in the presence of caffeine for 5 or 30 min followed by an additional culture in the absence of caffeine reduces polyspermic fertilization (Funahashi and Romar, 2004). Therefore, caffeine is useful for the improvement of IVF results, but its use during the gamete co-incubation should be considered.

## 5 Conclusions

The results of the present study indicate that both different males and the breed may influence the quality and fertility of frozen–thawed boar spermatozoa. The presence of caffeine in the IVF medium has a significant positive effect on the fertilization efficiency of low-quality microminipig spermatozoa. Moreover, an adequate length of sperm–oocyte co-incubation may improve IVF results.

## Data Availability

The data sets used in this paper are available from the corresponding author upon request.

## References

[bib1.bib1] Ahmad Z, Anzar M, Shahab M, Ahmad N, Andrabi SM (2003). Sephadex and sephadex ion-exchange filtration improves the quality and freezability of low-grade buffalo semen ejaculates. Theriogenology.

[bib1.bib2] Barbas JP, Mascarenhas RD (2009). Cryopreservation of domestic animal sperm cells. Cell Tissue Bank.

[bib1.bib3] Corcuera BD, Marigorta P, Sagues A, Saiz-Cidoncha F, Perez-Gutierrez JF (2007). Effect of lactose and glycerol on the motility, normal apical ridge, chromatin condensation and chromatin stability of frozen boar spermatozoa. Theriogenology.

[bib1.bib4] Cordova-Izquierdo A, Oliva JH, Lleo B, Garcia-Artiga C, Corcuera BD, Perez-Gutierrez JF (2006). Effect of different thawing temperatures on the viability, in vitro fertilizing capacity and chromatin condensation of frozen boar semen packaged in 5 ml straws. Anim Reprod Sci.

[bib1.bib5] Coy P, Martinez E, Ruiz S, Vazquez JM, Roca J, Matas C, Pellicer MT (1993). In vitro fertilization of pig oocytes after different coincubation intervals. Theriogenology.

[bib1.bib6] Dapino DG, Marini PE, Cabada MO (2006). Effect of heparin on in vitro capacitation of boar sperm. Biol Res.

[bib1.bib7] Do LT, Luu VV, Morita Y, Taniguchi M, Nii M, Peter AT, Otoi T (2015). Astaxanthin present in the maturation medium reduces negative effects of heat shock on the developmental competence of porcine oocytes. Reprod Biol.

[bib1.bib8] Fraser L, Strzezek J, Kordan W (2014). Post-thaw sperm characteristics following long-term storage of boar semen in liquid nitrogen. Anim Reprod Sci.

[bib1.bib9] Funahashi H, Day BN (1997). Advances in in vitro production of pig embryos. J Reprod Fertil Suppl.

[bib1.bib10] Funahashi H, Nagai T (2001). Regulation of in vitro penetration of frozen-thawed boar spermatozoa by caffeine and adenosine. Mol Reprod Dev.

[bib1.bib11] Funahashi H, Romar R (2004). Reduction of the incidence of polyspermic penetration into porcine oocytes by pretreatment of fresh spermatozoa with adenosine and a transient co-incubation of the gametes with caffeine. Reproduction.

[bib1.bib12] Funahashi H, Asano A, Fujiwara T, Nagai T, Niwa K, Fraser LR (2000). Both fertilization promoting peptide and adenosine stimulate capacitation but inhibit spontaneous acrosome loss in ejaculated boar spermatozoa in vitro. Mol Reprod Dev.

[bib1.bib13] Gangwar C, Saxena A, Patel A, Singh SP, Yadav S, Kumar R, Singh V (2018). Effect of reduced glutathione supplementation on cryopreservation induced sperm cryoinjuries in Murrah bull semen. Anim Reprod Sci.

[bib1.bib14] Gil MA, Alminana C, Cuello C, Parrilla I, Roca J, Vazquez JM, Martinez EA (2007). Brief coincubation of gametes in porcine in vitro fertilization: role of sperm:oocyte ratio and post-coincubation medium. Theriogenology.

[bib1.bib15] Kaneko N, Itoh K, Sugiyama A, Izumi Y (2011). Microminipig, a non-rodent experimental animal optimized for life science research: preface. J Pharmacol Sci.

[bib1.bib16] Larsson B, Rodriguez-Martinez H (2000). Can we use in vitro fertilization tests to predict semen fertility?. Anim Reprod Sci.

[bib1.bib17] Nagai T, Moor RM (1990). Effect of oviduct cells on the incidence of polyspermy in pig eggs fertilized in vitro. Mol Reprod Dev.

[bib1.bib18] Nagai T, Funahashi H, Yoshioka K, Kikuchi K (2006). Up date of in vitro production of porcine embryos. Front Biosci.

[bib1.bib19] Namula Z, Hirata M, Wittayarat M, Tanihara F, Thi Nguyen N, Hirano T, Nii M, Otoi T (2018). Effects of chlorogenic acid and caffeic acid on the quality of frozen-thawed boar sperm. Reprod Domest Anim.

[bib1.bib20] Nguyen VL, Somfai T, Nguyen TH, Nhung NT, Hong NT, Dat NT, Thinh NH, Van NK, Quyen DV, Chu HH, Son NT, Kikuchi K (2018). Optimization of the in vitro fertilization protocol for frozen epididymal sperm with low fertilization ability in Ban-A native Vietnamese pigs. Anim Sci J.

[bib1.bib21] Nishio K, Tanihara F, Nguyen TV, Kunihara T, Nii M, Hirata M, Takemoto T, Otoi T (2018). Effects of voltage strength during electroporation on the development and quality of in vitro-produced porcine embryos. Reprod Domest Anim.

[bib1.bib22] Parrish JJ (2014). Bovine in vitro fertilization: in vitro oocyte maturation and sperm capacitation with heparin. Theriogenology.

[bib1.bib23] Waterhouse KE, De Angelis PM, Haugan T, Paulenz H, Hofmo PO, Farstad W (2004). Effects of in vitro storage time and semen-extender on membrane quality of boar sperm assessed by flow cytometry. Theriogenology.

[bib1.bib24] Waterhouse KE, Hofmo PO, Tverdal A, Miller Jr RR (2006). Within and between breed differences in freezing tolerance and plasma membrane fatty acid composition of boar sperm. Reproduction.

[bib1.bib25] White IG (1993). Lipids and calcium uptake of sperm in relation to cold shock and preservation: a review. Reprod Fertil Dev.

[bib1.bib26] Wittayarat M, Kimura T, Kodama R, Namula Z, Chatdarong K, Techakumphu M, Sato Y, Taniguchi M, Otoi T (2012). Long-term preservation of chilled canine semen using vitamin C in combination with green tea polyphenol. Cryo Letters.

[bib1.bib27] Xu X, Pommier S, Arbov T, Hutchings B, Sotto W, Foxcroft GR (1998). In vitro maturation and fertilization techniques for assessment of semen quality and boar fertility. J Anim Sci.

[bib1.bib28] Yeste M (2016). Sperm cryopreservation update: Cryodamage, markers, and factors affecting the sperm freezability in pigs. Theriogenology.

